# NIR-Guided Coating Optimization of Omega-3 Fatty Acid Mini Soft Capsules with Pitavastatin and Ezetimibe

**DOI:** 10.3390/pharmaceutics16111374

**Published:** 2024-10-26

**Authors:** Hye-Ri Han, Ji Hoon Choi, Je Hwa Yoo, Jin-Hyuk Jeong, Sang-Beom Na, Ji-Hyun Kang, Dong-Wook Kim, Chun-Woong Park

**Affiliations:** 1 Department of Pharmacy, Chungbuk National University, Cheongju 28160, Republic of Korea; sky006112@gmail.com (H.-R.H.); zionncjh@kup.co.kr (J.H.C.); wpghk98@kdpharma.co.kr (J.H.Y.); jinddong92@gmail.com (J.-H.J.); sbna42@gmail.com (S.-B.N.); 2 Korea United Pharm R&D Center, Sejong 28530, Republic of Korea; 3 Kyung Dong Pharmaceutical Co., Ltd., Hwaseong 18631, Republic of Korea; 4 Institute of New Drug Development, and Respiratory Drug Development Research Institute, School of Pharmacy, Jeonbuk National University, Jeonju 54896, Republic of Korea; jhkanga@jbnu.ac.kr; 5 College of Pharmacy, Wonkwang University, Iksan 54538, Republic of Korea; pharmengin1@wku.ac.kr

**Keywords:** coating, near-infrared spectroscopy, process analytical technology, Omega-3 fatty acids

## Abstract

Background: This study aimed to optimize the coating process of Omega-3 fatty acid (OM3-FA) mini soft capsules containing the active pharmaceutical ingredients (APIs) pitavastatin and ezetimibe using near-infrared (NIR) spectroscopy for in-process monitoring. Cardiovascular disease treatments benefit from combining OM3-FA with lipid-lowering agents, but formulating such combinations in mini soft capsules presents challenges in maintaining stability and mechanical integrity. Methods: The coating process was developed using a pan coater and real-time NIR monitoring to ensure uniformity and quality. NIR spectroscopy enabled precise control of coating thickness, ensuring consistent drug distribution across the capsule surface. Results: The optimized process minimized OM3-FA oxidation and preserved the mechanical integrity of the capsules, as confirmed by texture analysis and in-vitro dissolution testing. This integration of NIR spectroscopy as a process analytical technology (PAT) significantly improved coating quality control, resulting in a stable and effective combination therapy for pitavastatin and ezetimibe in a mini soft capsule form. Conclusion: This approach offers an efficient solution for enhancing patient adherence in cardiovascular disease management. The application of NIR spectroscopy for real-time monitoring highlights its broader significance in pharmaceutical manufacturing, where it can serve as a versatile tool for ensuring product quality and optimizing production efficiency in diverse formulation processes. By incorporating NIR-based PAT, manufacturers can not only achieve product-specific improvements but also establish a foundation for continuous manufacturing and automated quality assurance systems, ultimately contributing to a more streamlined and robust production environment.

## 1. Introduction

Cardiovascular disease (CVD) is a leading cause of mortality worldwide [1]. Although pharmacological treatments have become the primary therapeutic approach for various cardiovascular conditions, there has been growing interest in the efficacy of natural health products for the prevention and treatment of CVD [2]. One natural product that has demonstrated favorable effects in CVD management is OM3-FA [3]. OM3-FA causes a dose-dependent reduction in serum triglyceride levels, with the effect being more pronounced in individuals with higher baseline triglyceride levels [4]. Observational and clinical trial data indicate that OM3-FA can reduce the risk of coronary heart disease-related mortality and nonfatal coronary events and suppress cardiac arrhythmias [5,6]. Many cardioprotective effects have been validated in human populations [7].

3-hydroxy-3-methylglutaryl-coenzyme A (HMG-CoA) reductase inhibitors, commonly known as statins, are first-line therapies for patients with elevated low-density lipoprotein (LDL) cholesterol levels [8]. However, statin monotherapy is often insufficient to achieve the recommended non-HDL cholesterol targets, and statins have only a moderate effect on lowering triglyceride levels [9]. Among available statins, pitavastatin is notable for its unique molecular structure and pharmacological properties. Like other statins, pitavastatin competitively inhibits HMG-CoA reductase, reduces cholesterol biosynthesis, and lowers LDL cholesterol levels [10]. Its fluorophenyl and cyclopropyl groups enhance LDL-C reduction even at low doses. The cyclopropyl group minimizes metabolism via the CYP3A4 enzyme, thereby reducing the risk of drug–drug interactions [11]. Additionally, pitavastatin is associated with a lower risk of new-onset diabetes mellitus than other statins, and studies have shown that it provides better glycemic control in type 2 diabetes patients than atorvastatin [12]. Another lipid-lowering agent, ezetimibe, can further reduce LDL cholesterol, either as a monotherapy or in combination with statins. Clinically, ezetimibe is used as an adjunct to statin therapy when additional lipid reduction or lower statin doses are needed [13,14].

Combining OM3-FA with other cardiovascular drugs offers substantial therapeutic benefits owing to their complementary effects; however, formulating them as a fixed-dose combination (FDC) presents significant challenges. The typical daily dose of OM3-FA is relatively high (usually between 2 to 4 g), and achieving this dose in a single soft capsule presents formulation difficulties due to the large size and liquid nature of OM3-FA. This high dose requirement further complicates formulation development, particularly in ensuring the uniformity and stability of the product. Although FDC therapies can improve patient adherence, reduce medication errors, and simplify treatment regimens, the development of such combinations, particularly OM3-FA, is complex [15]. OM3-FA is typically formulated as a liquid, making it difficult to integrate with solid medications such as statins or ezetimibe. This incompatibility often necessitates separate administration, further hindering patient compliance. Moreover, the large capsule size required to accommodate such high doses presents a challenge for patients who have difficulty swallowing, leading to poor compliance and reduced treatment efficacy. Despite the known therapeutic advantages of combining OM3-FA with other lipid-lowering agents, current formulations still struggle with patient adherence due to capsule size and stability issues, leaving a research gap in optimizing smaller, stable formulations that ensure efficacy and ease of use [16]. In addition, traditional softgel capsule technology often struggles to achieve consistent coating quality, particularly in formulations with multiple APIs. Variability in coating thickness can lead to uneven drug release, which negatively affects the therapeutic efficacy and safety of combination therapies. Furthermore, ensuring the stability of sensitive components like OM3-FA is particularly challenging, as conventional softgel manufacturing does not always provide adequate protection against oxidation or degradation during long-term storage. These limitations highlight the need for advanced coating technologies and monitoring systems that can ensure uniform coating application and drug release profiles [16]. OM3-FA are also highly prone to oxidation, compromising their stability and therapeutic efficacy. Furthermore, the characteristic fishy odor associated with OM3-FA products can be off-putting to patients, negatively impacting regular consumption [17,18]. These factors contribute to the overall complexity of developing effective and stable combination therapies in soft capsule form, where achieving uniform drug distribution and maintaining capsule integrity are key challenges.

To address these challenges, the development of smaller soft capsules with drug coatings can significantly improve patient compliance while maintaining the therapeutic efficacy of combination therapy [19,20]. Mini soft capsules coated with an API present several advantages for the formulation of combination therapies, particularly for compounds such as OM3-FA in conjunction with other cardiovascular drugs. One significant benefit is enhanced patient compliance resulting from the smaller capsule size, which makes swallowing easier, particularly for patients requiring long-term therapy [16,21]. The coating can also serve as a protective barrier, mitigating the oxidation of OM3-FA, which is a common issue due to its sensitivity to light and air. Furthermore, this approach enables the combination of liquid and solid dosage forms in a single capsule, streamlining administration and potentially reducing the overall pill burden for patients with complex therapeutic regimens [16,22]. By optimizing the coating process, mini soft capsules can ensure uniform distribution of API, providing consistent dosing and enhanced stability.

Despite these advantages, there are notable challenges associated with the API coating of mini soft capsules. One major issue is ensuring uniform application of the coating on each capsule, particularly when dealing with multiple APIs. The variability in coating thickness can result in inconsistent drug release profiles, compromising the therapeutic efficacy of the product [23]. The physical properties of OM3-FA combined with the need for heat-sensitive handling further complicate the coating process. Heat is often required for effective coating adhesion, but excessive heat can degrade OM3-FA, leading to loss of potency and stability [24]. Additionally, the low rigidity of small soft capsules makes them difficult to handle during manufacturing, thereby increasing the risk of capsule deformation or rupture under the mechanical stress of the coating process [25]. Furthermore, optimizing the adhesion of the coating to ensure stability without compromising the integrity of the capsule shell is a delicate balance that requires careful calibration of process parameters, such as temperature, coating solution viscosity, and spray rate. These challenges must be addressed through precise process control and real-time monitoring to ensure the quality of the final product.

NIR spectroscopy is a nondestructive analytical technique widely used in the pharmaceutical industry for the real-time monitoring and control of manufacturing processes. NIR operates by measuring the absorption of near-infrared light, typically between 780 and 2500 nm, by chemical compounds [26]. This absorption provides valuable information about the molecular composition and physical characteristics of the product, such as moisture content, drug concentration, and uniformity of the coatings. Owing to its rapid and noninvasive nature, NIR spectroscopy is ideal for ensuring product quality without the need for destructive testing [27]. NIR is often integrated as part of a broader framework known as PAT, a system encouraged by regulatory bodies, such as the FDA, to enhance process understanding and control. PAT involves the real-time monitoring of critical quality attributes during manufacturing with the aim of ensuring consistent product quality and enabling a shift from traditional batch testing to continuous manufacturing. By employing PAT tools, such as NIR spectroscopy, manufacturers can adjust parameters in real time, minimize batch-to-batch variability, and optimize the efficiency of the production process [28,29].

The primary objective of this study was to optimize the coating process of small OM3-FA soft capsules using NIR spectroscopy to ensure a uniform and stable coating of pitavastatin and ezetimibe. By employing a pan coater, which is commonly used in the pharmaceutical industry, this study aimed to apply a protective coating with minimal heat to prevent OM3-FA oxidation while enhancing process stability. The incorporation of NIR technology into the coating process enables real-time monitoring and precise quantification of drug distribution on the capsule surface, ensuring the quality and uniformity of the final product. This study is significant because it sought to reduce the capsule size while maintaining or improving the effectiveness of combination therapies, thereby improving patient compliance. Additionally, using NIR technology as a PAT, this study aims to enhance manufacturing efficiency, product stability, and scalability in the pharmaceutical industry. The potential to optimize production processes and ensure product quality through nondestructive real-time analysis is a valuable contribution to both patient care and industrial practices.

## 2. Materials and Methods

### 2.1. Materials

Pitavastatin calcium was supplied by Kyung Dong Pharmaceutical Co., Ltd. (USP, Gwacheon, Republic of Korea). Ezetimibe was provided by Ind-Swift Laboratories Ltd. (EP, Punjab, India). Livalo^®^ was provided by JW Pharmaceutical (Pitavastatin, Gwacheon, Republic of Korea), and Ezetrol^®^ was supplied by Organon Korea (Ezetimibe, Seoul, Republic of Korea). Seamless mini capsules were manufactured using facilities at Korea United Pharm (Omega-3 free fatty acid, Seoul, Republic of Korea). Two batches of capsules were received: OM-B-01, which consisted of normal beads with no weight variability, and OM-B-02, which consisted of defective beads with significant weight variability. Hydroxypropyl methyl cellulose (HPMC, Pharmacoat^®^ 645, Shin-Etsu Chemical Co., Ltd., Tokyo, Japan) was used as a coating polymer. Hydroxypropyl cellulose (HPC, Klucel ELF PHARM, Samin Chemical, Jeungpyeong-gun, Chungcheongbuk-do, Republic of Korea) was also incorporated as an alternative polymer to improve coating uniformity. Allura Red AC (Merck, Darmstadt, Germany) and Sunset Yellow FCF (Merck, Darmstadt, Germany) were added as colorants to enhance visual appeal. Titanium dioxide (TiO_2_, Freund Corporation, Tokyo, Japan) and talc (Reagent Chemicals, Daejung Chemicals & Metals, Siheung-si, Gyeonggi-do, Republic of Korea) were selected as inorganic colorants to enhance opacity and provide additional stability to the coating layer. Polyethylene glycol (PEG 4000, Extra Pure Reagent, Duksan Pure Chemicals, Ansan-si, Gyeonggi-do, Republic of Korea) was added as a plasticizer, and sodium lauryl sulfate (SLS, Chemical Pure, Daejung Chemicals & Metals, Siheung-si, Gyeonggi-do, Republic of Korea) was included as a surfactant to improve coating dispersion.

### 2.2. AI-Assisted Editing

This manuscript underwent an initial round of language refinement using OpenAI’s GPT-4 model, specifically for grammar and style improvement. This process helped enhance the clarity and readability of the English language in the manuscript. After the AI-based editing, a secondary round of professional review was conducted using Editage, a scientific editing service, to ensure that the manuscript met the journal’s language and formatting standards.

### 2.3. Preparation of Coated Capsules

The compositions of the protective and API layer coatings are listed in Table 1. The preparation method for each coating is as follows:

#### 2.3.1. Preparation of Protective Coating Solution

To prepare the protective layer coating solution, the colorant was dispersed in 70% ethanol using a homogenizer at 10,000 rpm for 10 min. The polymer was then added and stirred at 3000 rpm for 2 h. Finally, the coating solution was filtered through a 150 µm sieve.

#### 2.3.2. Coating Application for Protective Layer

Capsules were coated using a pan coater (Hana Addtech, Ansan, Republic of Korea). For formulations P1 to P2, the inlet temperature was set to 35 °C, the outlet temperature ranged from 28 to 30 °C, and the product temperature ranged from 21 to 25 °C. The air volume inside the coater was maintained below 1.2 m^3^/min, and the atomizing air pressure was set at 0.1 MPa. The flow rate of the coating solution was 3 mL/min, and the pan speed was set to 15 rpm. For formulations P3 to P5, the inlet temperature was set to 38 °C, the outlet temperature ranged from 32 to 36 °C, and the product temperature ranged from 30 to 33 °C. The other conditions were identical to those used for formulations P1 to P2.

#### 2.3.3. Preparation for API Coating Solution

A1 Coating: Pitavastatin was dissolved in distilled water and mechanically stirred at 3000 rpm for 30 min. Subsequently, the polymer was added, and the mixture was stirred at 3000 rpm for an additional 2 h.

A2 Coating: The colorant was dispersed in ethanol using a homogenizer at 10,000 rpm for 10 min. Ezetimibe was then added, and the mixture was mechanically stirred at 650 rpm for 30 min. The polymer, surfactant, and plasticizer were dissolved in distilled water and mechanically stirred at 650 rpm for 30 min. The two solutions were then combined and mechanically stirred at 650 rpm for 1 h. The final coating solution was filtered through a 150 μm sieve.

#### 2.3.4. API Coating Application

For the API coating formulation A1, the coating conditions were as follows: the inlet temperature was set to 55 °C, the outlet temperature ranged from 45 to 46 °C, and the product temperature ranged from 48 to 52 °C, ensuring proper adhesion of the coating layer. All other conditions were consistent with the previously mentioned settings for the protective coating layer.

For API coating formulation A2, the inlet temperature ranged from 45 to 50 °C, the outlet temperature ranged from 40 to 44 °C, and the product temperature ranged from 40 to 44 °C. The coating solution flow rate was adjusted to 2 mL/min to achieve the desired coating uniformity. The remaining parameters were identical to those used for formulation A1.

### 2.4. Evaluation of Protective Layer Coated Capsule

#### 2.4.1. Microscopic Examination of Capsule Appearance

The surfaces of the coated capsules were examined under an optical microscope (Siwon Optical Technology, Anyang, Republic of Korea) to identify potential defects or irregularities during the coating process.

#### 2.4.2. Contact Angle

The protective layer coating solution was applied to a glass slide. After coating, a drop of water less than 10 μL was placed on the coated glass slide. The shape of the water droplet was measured using a contact angle meter (Surface Electro Optics, Suwon, Republic of Korea). In the captured images, the glass slide surface touched by the hemispherical droplet was marked as a straight line, and the angle formed between the droplet and the straight line was calculated. The contact angle was calculated as the average of both ends of the droplet and the test was repeated thrice [30].

#### 2.4.3. Texture Analysis

The hardness of the coated capsules was measured using a texture analyzer (Micro Systems, Godalming, UK) equipped with a 36 mm diameter cylindrical probe (P/36R model). The probe was set to descend at a speed of mm/s, and the hardness of the capsules was determined at the point of maximum force at which rupture occurred [31].

#### 2.4.4. Scanning Electron Microscopy (SEM)

Non-coated and protective layer-coated capsules were cut using a scalpel. The cut capsules were pretreated by coating them twice with platinum. The prepared samples were observed using a scanning electron microscope (Carl Zeiss, Oberkochen, Germany). Non-coated capsules were observed at 100× magnification, whereas protective layer-coated capsules were observed at 300× magnification [32].

#### 2.4.5. Laser Confocal Microscopy (LCM)

The surfaces of the capsules were characterized using a laser confocal microscope (Keyence, Osaka, Japan). The sample surfaces were captured as 2D, 3D, and flattened 3D images. Observations were made on the corner of the capsule, with the observed sample area size being 500 × 700 µm. By analyzing the flattened 3D images, the influence of the protective layer coating on surface roughness was quantified. The arithmetic mean height (Sa) was calculated according to ISO 25178 standards using the Equation (1): [33,34]:

(1) $$Sa=\frac{1}{A}*\underset{A}{\iint }\left|Z\left(x,y\right)\right|dxdy$$

where Sa represents the average absolute value of the height deviation of each point from the reference plane over the entire surface area A. Function $Z\left(x,y\right)$ describes the height of the surface at each coordinate $\left(x,y\right)$. This measure provides a comprehensive assessment of the three-dimensional topography of the surface, offering a more detailed understanding of the surface roughness than two-dimensional parameters.

#### 2.4.6. Statistical Analysis

Statistical analysis was performed using one-way analysis of variance (ANOVA) to assess the differences among multiple groups. Post hoc comparisons were conducted using Dunnett’s multiple comparison test to compare each formulation with the reference group (such as the non-coated capsule). Significance levels were set at * *p* < 0.05, ** *p* < 0.01, and *** *p* < 0.005, indicating statistically significant differences between groups. Analyses were conducted using GraphPad Prism (GraphPad Software 8.4.2 (679), San Diego, CA, USA) to ensure a robust statistical evaluation of the data and accurate interpretation of the experimental results.

### 2.5. Evaluation of API Coated Capsule

#### 2.5.1. NIR Spectroscopy

NIR spectroscopy was used to quantify APIs on the surface of the coated capsules. NIR spectra were obtained using a MicroNIR 1700ES spectrometer (VIAVI Solutions, Chandler, AZ, USA) with the diffuse reflection method. Spectral analysis was performed using partial least-squares regression (PLSR) [35].

The measurement conditions were optimized by adjusting the scan count and sample size. The optimal scan count was determined by conducting five repeated measurements at various counts (50, 100, 150, 200, 250, and 300) and selecting the count with the smallest variance. The optimal number of samples per probe was determined by analyzing different sample quantities (4, 10, 16, and 20 capsules). NIR spectra were collected from each group and PLSR analysis was conducted to ensure proper probe coverage and consistent data acquisition.

Spectra collected under optimized conditions were preprocessed using the Savitzky-Golay 1st derivative (SG) and standard normal variate (SNV) to enhance data quality [36,37].

#### 2.5.2. In Vitro Release Study

In vitro release tests were conducted for both the test and reference formulations using the Vision C2 Classic 6 dissolution test system (Teledyne Hanson Research, Inc., Chatsworth, CA, USA) with a USP Apparatus II (paddle). The test methods for both formulations were sourced from the FDA Dissolution Methods Database.

For Formulation A1, using Livalo^®^ (pitavastatin 2 mg tablet) as the reference, the dissolution test was carried out in 900 mL of 0.05 M phosphate buffer at pH 6.8, maintained at 37 °C and 75 rpm. The sampling was performed at time points of 5, 10, 15, and 30 min.

For Formulation A2, using Ezetrol^®^ (ezetimibe 10 mg tablet) as the reference, the dissolution test was conducted in 500 mL of 0.45% sodium lauryl sulfate in 0.05 M acetate buffer at pH 4.5, maintained at 37 °C and 50 rpm. The sample was performed at time points of 10, 20, 30, and 45 min.

#### 2.5.3. High-Performance Liquid Chromatogram (HPLC) Analysis

HPLC, a technique widely used in the pharmaceutical industry for quantifying drugs and other substances, was performed using a Thermo U 3000 system (Thermo Fisher Korea, Seoul, Republic of Korea).

For the quantification of pitavastatin calcium and its related substances, reverse-phase HPLC was used with an Aegispak C18-L column (250 × 4.6 mm, 5 µm). The mobile phase consisted of two solutions: mobile phase A, a mixture of pH 3.8 acetate buffer and acetonitrile (ACN) in a 9:1 volume ratio, and mobile phase B, a mixture of ACN and distilled water in a 95:5 volume ratio. Both mobile phases were filtered through a 0.45 µm PVDF filter using a reduced pressure filtration device and degassed using a sonicator. The gradient conditions were set as follows: from 0 to 25 min, mobile phase A was held at 68% and mobile phase B at 32%. From 25 to 45 min, mobile phase A decreased from 68% to 10% and mobile phase B increased from 32% to 90%. From 45 to 55 min, mobile phase A was maintained at 10% and mobile phase B at 90%. At 55 min, the composition quickly reverted to 68% (A) and 32% (B) and was maintained until 60 min. The flow rate was set to 1.2 mL/min, the detection wavelength was 250 nm, and the injection volume was 20 µL.

For the quantification of ezetimibe, the HPLC analysis was conducted using a Kinetex C18 column (150 × 4.6 mm, 5 µm). The mobile phase was prepared by mixing tetrahydrofuran, acetonitrile, and the buffer in a 10:35:55 ratio. The buffer solution was prepared by dissolving 6.8 g of monobasic potassium phosphate in 1 L of water. The prepared mobile phase was filtered through a 0.45 µm PVDF filter using a reduced pressure filtration device and degassed with a sonicator. The flow rate was set to 1 mL/min, the detection wavelength was 232 nm, and the injection volume was 30 µL.

## 3. Results and Discussion

### 3.1. Evaluation of Protective Layer-Coated Capsules

#### 3.1.1. Microscopic Examination of Capsule Appearance

Pan coating was conducted on each protective layer coating formulation to evaluate their performance and identify any process-related defects. Following the coating process, the capsules were observed under an optical microscope to assess the surface characteristics and to investigate potential defects, allowing for subsequent process improvements. Since formulations P1 to P3 did not utilize metal salt colorants, Allura Red AC or Brilliant Yellow pigments were used as alternatives.

**Figure 1:**
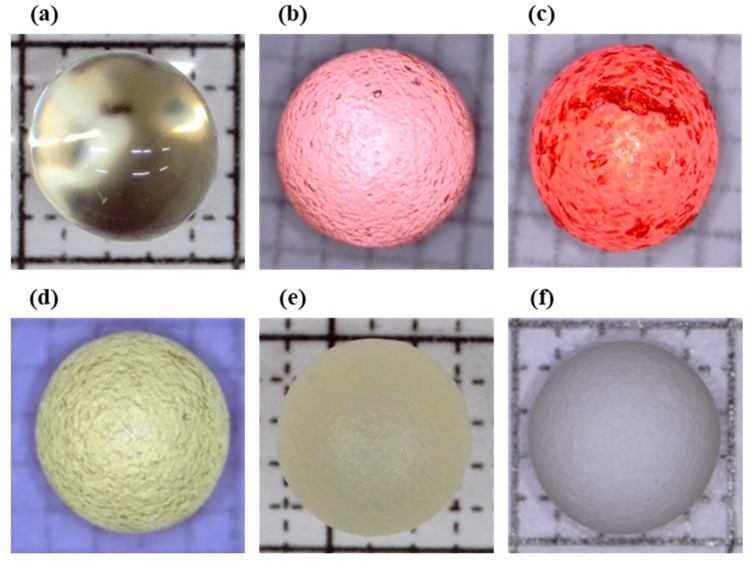
Optical microscopic images of capsule appearance. (**a**) Non-coated capsule; (**b**) P1-coated capsule; (**c**) P2-coated capsule; (**d**) P3-coated capsule; (**e**) P4-coated capsule; (**f**) P5-coated capsule.

Figure 1a shows a seamless mini capsule observed under an optical microscope prior to the coating process, displaying a smooth and uniform surface. In Figure 1b, the P1 formulation, which utilized only HPC as the polymer, exhibited aggregation of the colorant. This failure in the process is likely attributable to the sticky nature of HPC, leading to adhesion issues. As a result, the polymer was switched from HPC to HPMC in subsequent formulations. Figure 1c depicts a capsule with the P2 formulation, which did not encounter adhesive failures during the coating process. However, the film layer was poorly deposited, likely due to the low solids content in the coating dispersion. To address this, the solids content of the coating solution was increased from 3% to 6%. In Figure 1d, corresponding to the P3 formulation where the polymer ratio was adjusted to 8:7, the samples showed no signs of agglomeration in the pan coater, indicating successful coating. Although Yellow pigments were used in the P3 formulation, the coated capsules appeared light in color under the microscope. This phenomenon could be due to partial absorption of the pigment into the polymer matrix, reducing the overall color intensity. The protective layer serves as a barrier, enhancing the adhesion of the API coating while minimizing the risk of coating defects. This interaction ensures uniform coating distribution and prevents issues such as adhesion-related aggregation during the coating process, contributing to a smooth and defect-free surface. The optimized protective layer formulation with a well-balanced polymer ratio helps achieve a uniform API layer, which is critical for maintaining the mechanical stability of the final capsule product.

For formulations P4 and P5, metal salt colorants were incorporated into the process while maintaining the polymer ratio. Figure 1e shows the P4-coated capsule, which demonstrated a consistent film surface. The addition of talc as an anti-sticking agent minimized clumping and improved film adhesion. Similarly, the inclusion of metal salt colorants in the P4 formulation further strengthened the integrity of the protective layer, providing a stable base for subsequent API coating. Figure 1f displays the P5-coated capsule, which included TiO_2_ as an additional colorant. TiO_2_ not only enhanced the opacity of the film but also contributed to a smoother surface texture, minimizing visible defects. This resulted in a more uniform coating layer, facilitating even drug distribution and reducing the risk of localized drug accumulation.

#### 3.1.2. Contact Angle

Soft gelatin capsules are inherently vulnerable to water because of their composition, making them susceptible to damage when exposed to aqueous environments [38]. To address this challenge, a protective coating layer was applied to enhance the water resistance of the capsules, particularly when followed by aqueous API coating. This study evaluated the waterproofing properties of a protective coating layer to mitigate the potential issues associated with the application of aqueous coatings on water-sensitive soft gelatin capsules.

**Figure 2:**
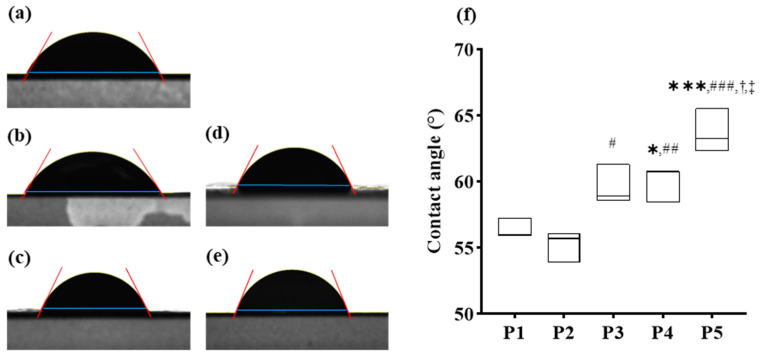
Observation and Contact Angle Analysis of Water Droplets on Formulation-Specific Slides. (**a**) P1; (**b**) P2; (**c**) P3; (**d**) P4; (**e**) P5. (**f**) Comparison of contact angles of water droplets on different formulation-specific slides (P1, P2, P3, P4, and P5), highlighting statistically significant differences among the formulations. Ordinary one-way ANOVA followed by Dunnett’s multiple comparison test was performed to compare each formulation against P1. * *p*-value < 0.05 and *** *p*-value < 0.005 indicate statistically significant differences compared with P1; # *p*-value < 0.05, ## *p*-value < 0.01, and ### *p*-value < 0.005 indicate differences compared with P2; † *p*-value < 0.05 compared with P3; ‡ *p*-value < 0.05 compared with P4.

Figure 2 shows the contact angles of water droplets on the surfaces of various protective layer formulations. Statistically significant differences were observed among the formulations, as indicated by the ANOVA results shown in Figure 2. Formulations P1 and P2 exhibited contact angles of 56.39 ± 0.75° and 55.24 ± 1.19°, respectively, with no statistically significant difference between them, indicating similar waterproofing properties when using hydroxypropyl cellulose and hydroxypropyl methyl cellulose.

Formulation P2 displayed a higher contact angle of 59.59 ± 1.50°, showing statistically significant improvement in water resistance compared to P1 (* *p* < 0.05). Formulation P4 demonstrated a contact angle of 59.97 ± 1.34°, which was significantly higher than P1 and P2 (** *p* < 0.01), indicating enhanced waterproofing. The highest contact angle was observed in formulation P5 at 63.71 ± 1.64°, which was significantly greater than all other formulations (*** *p* < 0.005), indicating superior water resistance.

These results indicate that the combination of metal salt colorants with polymers significantly enhances the waterproofing properties of the protective layer compared to the polymers alone. TiO_2_ provided the highest level of waterproofing among the metal salt colorants, as evidenced by its increased contact angles. This enhanced waterproofing is crucial for maintaining the structural integrity of water-sensitive soft gelatin capsules during subsequent application of aqueous API coatings, thereby minimizing potential process disruptions and ensuring consistent product quality.

The improved waterproofing properties demonstrated by the higher contact angles in formulations P3, P4, and P5 suggest that careful selection of hydrophobic components can lead to significant advancements in protective coating technology for soft gelatin capsules. This is particularly important in pharmaceutical applications, where capsules must maintain their integrity in environments that may otherwise compromise their physical and chemical stabilities. For instance, P5’s use of TiO_2_ not only increased water resistance but also provided a robust barrier against moisture, which is critical for preserving the efficacy of moisture-sensitive APIs.

#### 3.1.3. Texture Analysis

**Figure 3:**
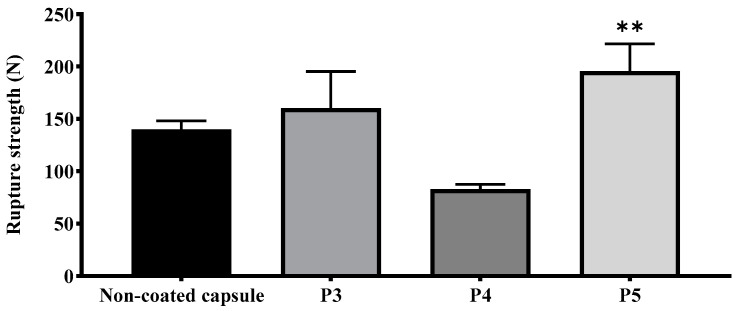
Capsule hardness, depending on coating formulation. Ordinary one-way ANOVA followed by Dunnett’s multiple comparison test was performed to compare each formulation against the non-coated capsule. ** *p*-value < 0.005 indicate statistically significant differences compared with the non-coated capsule.

Soft gelatin capsules are characterized by low durability, which can lead to process disruption during coating. Therefore, a protective coating layer was applied to enhance the capsule rigidity. Texture analysis of the capsules was conducted to evaluate the rupture strength as a function of the coating formulation and time. As shown in Figure 3, rupture strength varied significantly among the different formulations. The non-coated capsule exhibited a rupture strength of approximately 150 N. Formulation P3 showed a similar strength, with no significant difference (mean difference: −20.27, *p* = 0.5577). However, P4 formulations exhibited significantly lower rupture strengths compared to the non-coated capsule, with values of 79.56 N, respectively (*p* < 0.05).

Formulation P5 demonstrated the highest rupture strength among all tested samples, with a value of 194.50 N, which was significantly higher than the non-coated capsule (mean difference: −55.43, *p* = 0.0038). This indicates that the protective layer coating in P5 enhanced the mechanical stability of the capsules, suggesting improved performance under stress conditions.

#### 3.1.4. SEM

**Figure 4:**
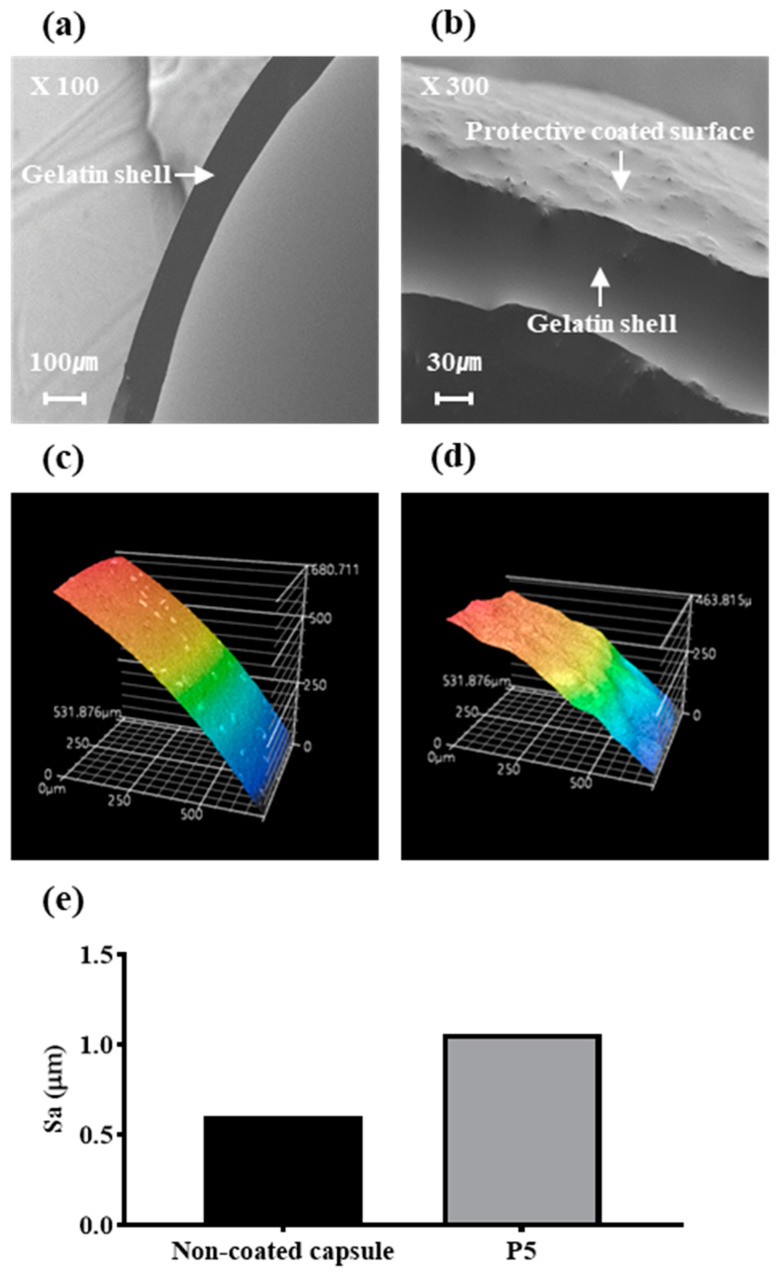
Images of capsule surfaces. (**a**) SEM image of a non-coated soft gelatin capsule; (**b**) SEM image of a capsule coated with the formulation P5; (**c**) LCM image of non-coated soft gelatin capsule; (**d**) LCM image of a capsule coated with the formulation P5; (**e**) arithmetic mean height of the scaled surface.

Cross-sectional images of the soft gelatin capsules were obtained using SEM. Figure 4a shows the cross section of a non-coated soft gelatin capsule. The capsule wall is approximately 100 µm thick with a smooth surface, characteristic of uncoated gelatin capsules.

Figure 4b illustrates the cross section of a soft gelatin capsule coated with a protective layer from formulation P5. The cross-sectional thickness of the coated capsules was similar to that of the non-coated capsules, indicating that there was no significant change in thickness due to gelatin modification during the coating process. However, unlike the smooth surface observed in the non-coated capsules, the P5-coated capsules exhibited a rough and uneven surface. This roughness was attributed to the application of a protective coating layer, which altered the surface morphology of the capsule.

#### 3.1.5. LCM

The surface roughness of the non-coated capsules and protective layer-coated soft gelatin capsules (formulation P5) was evaluated using LCM. Figure 4c,d show 3D images of the surfaces of the non-coated and P5-coated capsules, respectively. The arithmetic mean height (Sa) was calculated from the flattened 3D images to quantify surface roughness.

According to Figure 4e, the Sa value of the non-coated capsule was 0.5650 µm, indicating a relatively smooth surface typical of gelatin capsules without additional coatings. In contrast, the P5-coated capsule exhibited a significantly increased Sa value of 1.125 µm, demonstrating a rougher surface due to the application of the protective layer coating. This rougher surface indicates that the protective coating changes the surface morphology, making it more textured, which may improve the adhesion of subsequent API coatings.

A comparison between the non-coated and P5-coated capsules highlighted that the protective layer coating significantly modified the capsule surface, making it rougher and potentially more suitable for effective drug deposition.

### 3.2. Evaluation of API Layer Coated Capsules

#### 3.2.1. Impact of Capsule Weight Variability on Endpoint Prediction in API Coating Processes

The API coating processes were conducted separately for A1 (pitavastatin) and A2 (ezetimibe). A batch of normal soft gelatin capsules with minimal weight variability was used for coating A1, whereas a batch with significant variability with defective capsules was used for coating A2. This approach aimed to assess the effect of weight variability on endpoint prediction during drug quantification using weight gain.

**Figure 5:**
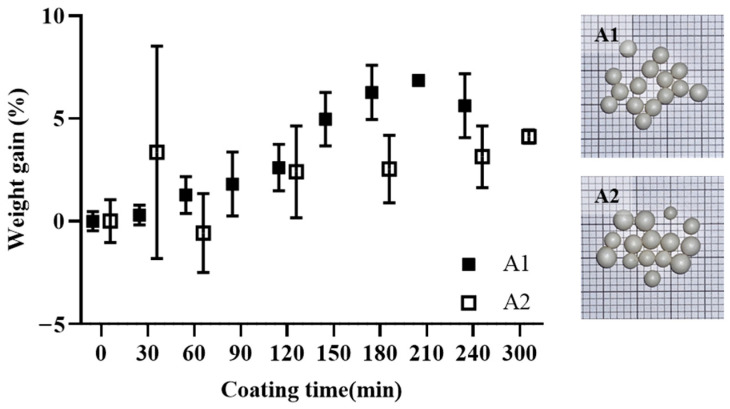
Percentage of weight gain according to API coating time for A1 and A2.

The theoretical endpoint for A1 was approximately 5% (*w*/*w*%) weight gain, whereas that for A2 was approximately 9% (*w*/*w*%). Figure 5 shows the percentage of weight gain over time during the coating process. A1 demonstrated relatively consistent weight gain, whereas A2 displayed irregular weight gains with greater variability. Uniform weight gain is an important indicator of the coating process. This allows for the indirect prediction of the coating endpoint and serves as a way to verify the absence of process-related issues [39].

**Figure 6:**
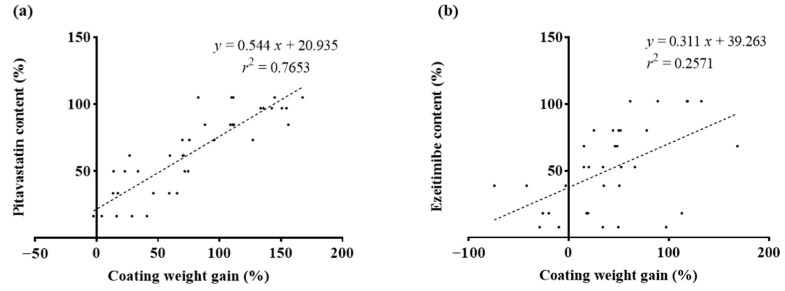
Scatterplot of drug content (%) by HPLC and coating weight gain (%). (**a**) A1; (**b**) A2.

Figure 6 illustrates the correlation between weight gain and the actual drug content quantified via HPLC. A1 showed a correlation coefficient of 0.7653 between weight gain and pitavastatin content, which was higher compared to the correlation coefficient of 0.2571 for A2, between weight gain and ezetimibe content. This suggests that weight gain was more reliable for predicting the endpoint in A1 than in A2, highlighting the challenges of using weight-based predictions under conditions with significant variability.

#### 3.2.2. NIR Spectroscopy

##### Optimization of NIR Measurement

**Figure 7:**
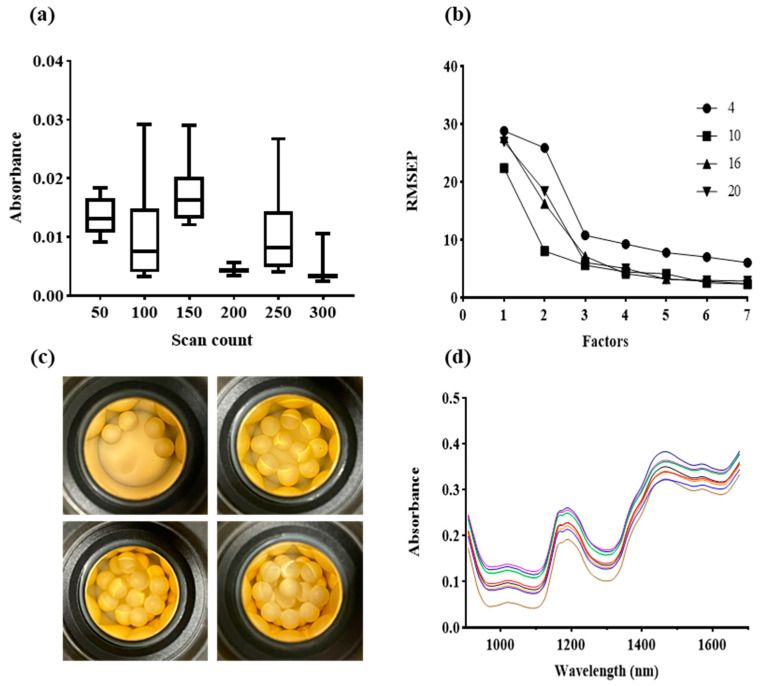
Near-infrared spectroscopy process settings. (**a**) Scan count. (**b**) Count factor analysis. (**c**) Images of samples placed in the probe for NIR spectroscopy. From the top left: 4 samples; top right: 10 samples; bottom left: 16 samples; and bottom right: 20 samples. (**d**) API absorbance spectra.

To improve the endpoint prediction under variable conditions, NIR spectroscopy was introduced as a PAT method for nondestructive in-process monitoring that adapts to diverse conditions, including normal and defective capsules with high weight variability. Figure 7 illustrates the optimization of the NIR settings. Figure 7a shows the optimization of the scan counts using a boxtail plot. Although a scan count of 300 yielded the lowest standard deviation, 200 scans were selected because of the sufficiently low variance and standard deviation, balancing efficiency and accuracy.

Figure 7b shows the root mean square error of prediction (RMSEP) values for different sample counts analyzed using PLSR. An optimized scan count of 200 was used, and the smallest RMSEP value was obtained when 10 samples were measured until the number of factors reached three, indicating the best model fit. Figure 7c further confirms these results; the top-left image shows uneven coverage with four samples, the top-right image shows uniform probe coverage with 10 samples, and the bottom-left and bottom-right images demonstrate stacking issues with 16 and 20 samples, respectively. Such uneven coverage leads to inconsistent measurements, emphasizing the importance of selecting an optimal sample count for reliable NIR measurements.

Figure 7d shows the absorbance spectra with a consistent increase in absorbance correlating with increased coating amounts, confirming that NIR measurements effectively monitored the coating progression under the optimized conditions of 200 scans and 10 samples.

##### Predictive Control of Coating Quality Using NIR Spectroscopy

The coating process was validated using NIR spectroscopy combined with PLSR models to ensure the accuracy and reliability of in-process monitoring. NIR spectroscopy is a nondestructive real-time analytical method that allows continuous monitoring of the coating process without interrupting the production line [40]. This approach is particularly advantageous in pharmaceutical manufacturing, where maintaining a consistent coating quality is crucial for dosage uniformity and drug release profiles [40].

**Figure 8:**
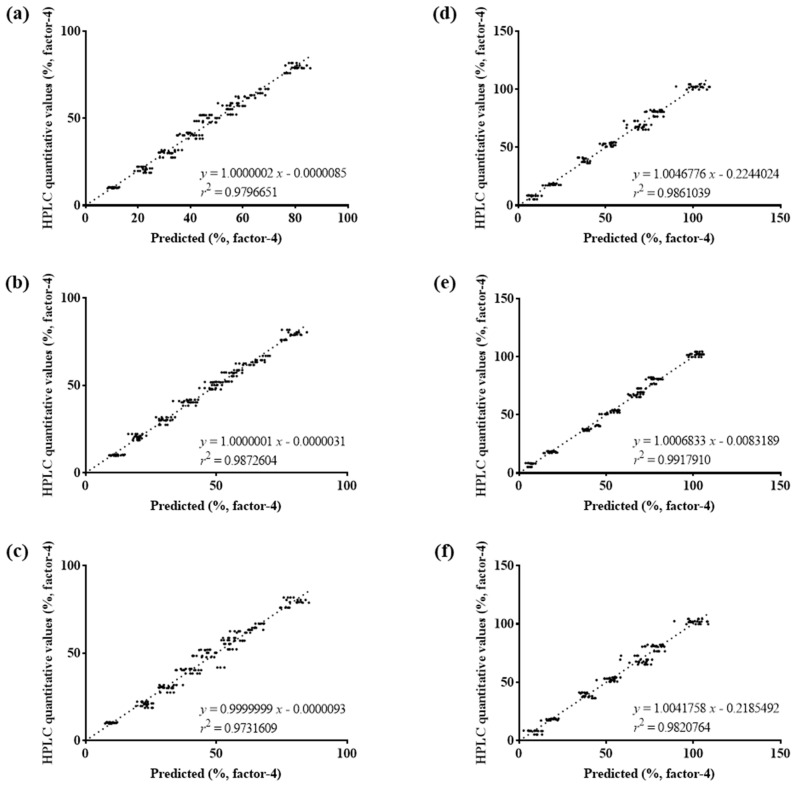
Correlation between HPLC quantification value and predicted (PLSR model). (**a**) SG of A1; (**b**) SNV of A1; (**c**) SG and SNV of A1; (**d**) SG of A2; (**e**) SNV of A2; (**f**) SG and SNV of A2.

Preprocessing transformed the NIR measurement results. SG and SNV were used as preprocessing algorithms [41]. Figure 8 illustrates the strong correlation between the predicted values from the PLSR models and the reference values obtained from the HPLC quantification of both pitavastatin (A1) and ezetimibe (A2). The SNV model consistently demonstrated the highest correlation coefficients, with R^2^ values of 0.9918 for A1 and 0.9821 for A2, indicating that SNV preprocessing significantly enhanced the predictive capability of the NIR models. This finding underscores the high suitability of SNV-enhanced NIR models for the real-time monitoring of coating quality, as they provide accurate and reliable predictions that are critical for maintaining product quality.

In contrast, Table 2 shows the correlation between weight gain predictions and the actual drug content quantified by HPLC, highlighting the limitations of using weight gain alone as a predictive measure. The lower correlation coefficients in Table 2, with R^2^ values of 0.7653 for A1 and 0.2571 for A2, indicate that weight gain predictions do not reliably reflect actual drug content, especially under variable conditions. This comparison illustrates that NIR spectroscopy, particularly when enhanced with SNV preprocessing, provides superior accuracy and reliability in predicting coating quality. Moreover, a quantitative method using NIR spectroscopy demonstrated that it was unaffected by weight variability, making it a more robust solution for real-time quality control in the coating process, ensuring consistent product quality even under variable conditions [42].

Table 3 presents a detailed evaluation of the precision, accuracy, and difference of the SNV model across various coating times for both A1 and A2 formulations. The difference values indicate how closely the predicted values align with the actual HPLC quantification, averaging between 95.92 and 100.98 for both A1 and A2. The data show that the SNV model provides predictions with precision values ranging from 2.01 to 10.53, and accuracy values ranging from 2.85 to 10.53, demonstrating strong alignment with the reference measurements. The consistent precision, accuracy, and difference across different coating times indicate that NIR spectroscopy, when used with the SNV model, can effectively monitor coating consistency, detect deviations in real time, and adjust process parameters accordingly [43].

The validation demonstrates that the SNV-enhanced NIR model can reliably track the coating process under variable conditions, offering a robust solution for real-time quality control. This method reduces the risk of coating defects, such as uneven thickness or incomplete coverage, which can affect drug release and efficacy [44]. In-process monitoring using NIR ensures the production of high-quality pharmaceutical products by maintaining stringent control over critical process parameters [44].

#### 3.2.3. In Vitro Dissolution Test

**Figure 9:**
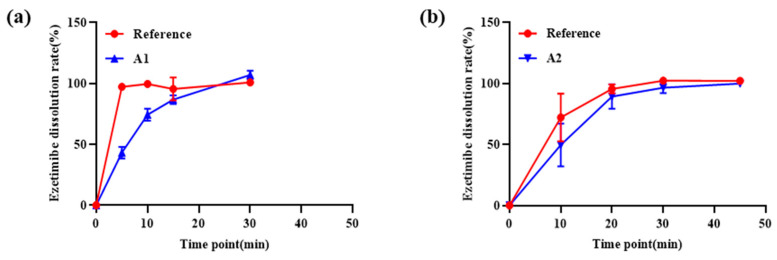
Comparative dissolution testing. (**a**) Pitavastatin dissolution test results at pH 6.8 for A1 formulation and reference product; (**b**) ezetimibe dissolution test results at pH 4.5 for A2 formulation and reference product.

In vitro dissolution testing was performed to evaluate the release profiles of the formulations compared with those of the reference products. Figure 9 shows the comparative dissolution results for the A1 (pitavastatin) and A2 (ezetimibe) formulations.

For A1, the dissolution test in pH 6.8 phosphate buffer demonstrated a dissolution profile similar to the reference product, Livalo^®^. The dissolution data indicated a rapid initial release, achieving over 80% release within 10 min, followed by a plateau. A2 dissolution in pH 4.5 acetate buffer also exhibited release characteristics similar to the reference product, Ezetrol^®^, with initial rapid release reaching approximately 70% within 20 min before slowing.

Mathematical modeling was applied to understand the release kinetics. For pitavastatin, the first-order kinetics model provided the best fit (R^2^ = 0.9950), suggesting that the release rate was dependent on the amount of the remaining drug. The Higuchi model (R^2^ = 0.9766) also indicated diffusion-controlled release. Conversely, ezetimibe release kinetics were best described by the Hixon-Crowell model (R^2^ = 0.9721), indicating that the release is influenced by the erosion and dissolution of the coating. The first-order model (R^2^ = 0.9691) also showed a good fit, reflecting the dependence of the release rate on the drug concentration.

The findings of this study emphasize the importance of the formulation in achieving the desired dissolution profiles, supporting the applicability of these models in predicting the drug release behavior in both immediate-release formulations. Modeling drug release from delivery systems, especially HPMC, is widely used in pharmaceutical studies to ensure optimal release profiles [45]. Modeling and comparison of dissolution profiles are crucial for predicting the behavior of formulations under various conditions [46]. These findings support the role of kinetic modeling in the design of drug delivery systems [47].

## 4. Discussion

The protective coating formulations evaluated in this study demonstrated varying degrees of water resistance and mechanical stability. Figure 1 shows the surface characteristics of non-coated and coated capsules, highlighting differences among the formulations. The P1 formulation, which used HPC as the polymer, exhibited aggregation of the colorant, likely due to the sticky nature of HPC leading to adhesion issues during the coating process. To address this, the polymer was switched to HPMC in the P2 formulation.

While P2 formulation did not encounter the same adhesion issues, it suffered from poor film deposition, likely due to the low solids content in the coating solution. To resolve this, the solids content was increased from 3% to 6% in subsequent formulations. In Figure 1d, the P3 formulation, which adjusted the polymer ratio to 8:7, demonstrated improved coating with no signs of agglomeration, indicating successful deposition.

The contact angle measurements shown in Figure 2f illustrate significant improvements in water resistance with the addition of metal salt colorants. The incorporation of TiO₂ in P5 led to the highest contact angle, suggesting improved hydrophobicity, consistent with previous studies [30,31].

Texture analysis, as presented in Figure 3, revealed that the mechanical strength of the P5-coated capsules was significantly higher than the non-coated capsules, indicating enhanced durability under stress. This result aligns with prior findings on the benefits of protective layers in maintaining structural integrity during coating [21,24].

SEM and LCM images in Figure 4 showed that the P5 formulation created a rougher surface, with an increase in arithmetic mean height (Sa), enhancing adhesion between the API and the capsule surface [33].

The variability in weight gain during API coating processes, as shown in Figure 5, highlights challenges in predicting the endpoint accurately. A1, coated with pitavastatin, exhibited consistent weight gain, while A2, coated with ezetimibe, showed greater variability due to defective capsules [13,14]. This reinforces the need for alternative monitoring techniques beyond weight gain alone [23,26].

To overcome these challenges, NIR spectroscopy was introduced as a PAT for real-time monitoring. Optimized NIR settings improved prediction accuracy, with PLSR models showing superior performance when combined with SNV preprocessing, as illustrated in Figure 8 [27,28]. These models achieved high R² values, confirming the reliability of NIR spectroscopy for in-process monitoring [36,37].

The in vitro dissolution testing, detailed in Figure 9, confirmed that both A1 and A2 formulations exhibited release profiles comparable to their reference products (Livalo® for pitavastatin and Ezetrol® for ezetimibe), supporting effective drug delivery [5]. Mathematical modeling revealed that pitavastatin followed a first-order release model, while ezetimibe was best described by the Hixon-Crowell model, reflecting the influence of coating thickness and erosion on release profiles [45,46].

Despite the successful application of NIR spectroscopy for real-time monitoring and optimization of the coating process, several limitations need to be addressed in future research. First, this study focused on a specific combination of APIs and coating materials. Although the results demonstrated the effectiveness of the proposed NIR model, the applicability of this method to other APIs or excipients has not been fully validated. Further research should investigate the robustness of the NIR model across a broader range of formulations, capsule sizes, and coating types to enhance its generalizability in pharmaceutical manufacturing.

Additionally, while the study successfully incorporated NIR spectroscopy as a PAT tool for quality control, other potential sources of variability, such as environmental conditions (e.g., humidity and temperature), were not explicitly controlled. Future studies should explore integrating NIR spectroscopy with other PAT tools to comprehensively monitor these variables in real time, thereby ensuring more robust control over the coating process.

Finally, the mechanical properties of the coated capsules were not extensively evaluated in this study. As the coating layer significantly influences the stability and handling of the capsules during downstream processes, future research should focus on understanding the impact of different coating formulations on capsule integrity and mechanical properties under various storage and transport conditions.

In conclusion, expanding the scope of NIR spectroscopy to encompass a wider range of formulations and incorporating additional monitoring tools will further validate its effectiveness as a versatile and reliable tool for in-process quality control in pharmaceutical manufacturing.

## 5. Conclusions

This study successfully established a process for coating seamless mini capsules using a pan coater. The process was optimized to ensure consistent coating application even in conditions with significant batch variability, which demonstrates the feasibility of applying NIR spectroscopy for process monitoring under various conditions. The protective coating significantly enhanced water resistance, which is crucial for maintaining the structural integrity of capsules during subsequent aqueous API coating processes. This approach effectively mitigates the challenges associated with aqueous coatings on water-sensitive soft gelatin capsules, ensuring uniform drug distribution and minimizing potential process disruption.

The results showed that combining metal salt colorants, particularly titanium dioxide with polymers, significantly improved the waterproofing properties of the protective layer compared with the use of polymers alone. Statistical analysis confirmed that the formulations with enhanced water resistance exhibited higher contact angles, demonstrating superior performance in protecting capsules from aqueous exposure.

Furthermore, the in vitro dissolution tests revealed distinct release kinetics for pitavastatin and ezetimibe, with each drug fitting specific kinetic models. Pitavastatin release was best described by first-order kinetics, indicating a concentration-dependent release, whereas ezetimibe release followed the Hixson–Crowell model, suggesting a combination of dissolution and erosion mechanisms. These findings underscore the importance of tailored formulation strategies for achieving desired drug release profiles.

In conclusion, the integration of NIR spectroscopy not only allows for in-process monitoring but also facilitates coating quality analysis under various challenging conditions, such as batch variability and process anomalies. This nondestructive, efficient, and adaptable solution ensures the production of high-quality pharmaceutical products. These findings align with previous studies where NIR spectroscopy was successfully utilized as a PAT tool for real-time monitoring of coating processes, demonstrating enhanced precision and quality control under variable manufacturing conditions [43,44]. The consistent results from our study and others suggest that the integration of NIR into pharmaceutical manufacturing can reliably predict coating uniformity and detect deviations, thereby reducing the risk of batch rejections [42]. Future research should extend the applicability of this method to a wider range of formulations and coating materials, further confirming its role in pharmaceutical manufacturing.

## Figures and Tables

**Table 1:** Formulation of protective layer coating and API layer coating.

(%)	Protective Layer					API Layer	
Code	P1	P2	P3	P4	P5	A1	A2
Pitavastatin						1.0	
Ezetimibe							4.0
Hydroxypropyl cellulose	95.5		52.8	40.0		51.0	
Hydroxypropyl methyl cellulose		99.0	46.8	35.0	40.0	48.0	88.0
Talc				25.0	35.0		3.0
Titanium dioxide					25.0		
Sodium lauryl sulfate							1.0
Allura Red AC	0.5	1.0					
Sunset yellow FCF			0.5				
Polyethylene glycol							5
Solid content	3.0	3.0	6.0	6.0	6.0	6.0	6.0

**Table 2:** Correlation coefficients between HPLC quantification values and coating weight gain compared to predicted values using PLSR models with different preprocessing methods.

Code	HPLC Quantification and Coating Weight Gain	HPLC Quantification and Predicted		
		SG	SNV	SG and SNV
A1	0.7653	0.9767	0.9873	0.9732
A2	0.2571	0.9861	0.9918	0.9821

**Table 3:** Precision, accuracy, and difference of SNV model for in-process monitoring of coating quality.

Code	Coating Time (min)	HPLC Quantification (% ± SD)	Predicted (% ± SD)	Precision (Average ± SD)	Accuracy (Average ± SD)	Difference (Average ± SD)
A1	60	30.14 ± 1.43	31.28 ± 2.37	5.94 ± 2.38	5.48 ± 4.14	99.30 ± 2.25
	120	56.15 ± 2.25	55.72 ± 2.39	2.43 ± 0.57	4.87 ± 1.59	99.80 ± 2.71
	160	79.22 ± 1.95	79.85 ± 3.01	2.01 ± 0.500	2.85 ± 2.04	100.98 ± 2.81
A2	60	18.18 ± 0.58	18.95 ± 2.44	10.53 ± 2.00	6.22 ± 4.43	100.18 ± 6.84
	120	38.76 ± 1.81	38.79 ± 2.55	3.73 ± 1.14	7.63 ± 5.90	95.92 ± 7.80
	180	52.77 ± 1.31	52.91 ± 2.75	3.52 ± 1.88	2.77 ± 2.53	102.43 ± 5.07
	240	68.44 ± 2.57	69.62 ± 4.52	5.21 ± 2.12	5.86 ± 3.87	99.92 ± 2.74
	300	80.24 ± 1.89	78.64 ± 3.47	3.77 ± 1.06	3.77 ± 1.65	100.10 ± 2.17
	400	102.06 ± 1.45	101.04 ± 4.28	3.35 ± 0.76	3.07 ± 1.11	99.38 ± 1.64

## Data Availability

The datasets used in this study are available from the authors upon request.

## References

[1] Genest J., McPherson R., Frohlich J., Anderson T., Campbell N., Carpentier A., Couture P., Dufour R., Fodor G., Frencis G.A. (2009). 2009 Canadian Cardiovascular Society/Canadian guidelines for the diagnosis and treatment of dyslipidemia and prevention of cardiovascular disease in the adult–2009 recommendations. Can. J. Cardiol..

[2] Blackwood D., LaVallee R.K., Busaidi A.A., Jassal D.S., Pierce G.N. (2015). A randomized trial of the effects of ezetimibe on the absorption of omega-3 fatty acids in cardiac disease patients: A pilot study. Clin. Nutr. ESPEN.

[3] Paschos G.K., Magkos F., Panagiotakos D.B., Votteas V., Zampelas A. (2007). Dietary supplementation with flaxseed oil lowers blood pressure in dyslipidaemic patients. Eur. J. Clin. Nutr..

[4] Psota T.L., Gebauer S.K., Kris-Etherton P. (2006). Dietary omega-3 fatty acid intake and cardiovascular risk. Am. J. Cardiol..

[5] Yokoyama M., Origasa H., Matsuzaki M., Matsuzawa Y., Saito Y., Ishikawa Y., Oikawa S., Sasaki J., Hishida H., Itakura H. (2007). Effects of eicosapentaenoic acid on major coronary events in hypercholesterolaemic patients (JELIS): A randomised open-label, blinded endpoint analysis. Lancet.

[6] Leaf A. (2007). Omega-3 fatty acids and prevention of arrhythmias. Curr. Opin. Lipidol..

[7] Patade A., Devareddy L., Lucas E.A., Korlagunta K., Daggy B.P., Arjmandi B.H. (2008). Flaxseed reduces total and LDL cholesterol concentrations in Native American postmenopausal women. J. Womens Health.

[8] Rosenson R.S. (2017). Statins: Actions, side effects, and administration. Up Date Database Topic.

[9] Davidson M.H., Maki K.C., Pearson T.A., Pasternak R.C., Deedwania P.C., McKenney J.M., Fonarow G.C., Maron D.J., Ansell B.J., Clark L.T. (2005). Results of the national cholesterol education (NCEP) program evaluation project utilizing novel E-technology (NEPTUNE) II survey and implications for treatment under the recent NCEP writing group recommendations. Am. J. Cardiol..

[10] Saito Y. (2011). Pitavastatin: An overview. Atheroscler. Suppl..

[11] Catapano A.L. (2010). Pitavastatin–pharmacological profile from early phase studies. Atheroscler. Suppl..

[12] Seo W.W., Seo S.I., Kim Y., Yoo J.J., Shin W.G., Kim J., You S.C., Park R.W., Park Y.M., Kim K.J. (2022). Impact of pitavastatin on new-onset diabetes mellitus compared to atorvastatin and rosuvastatin: A distributed network analysis of 10 real-world databases. Cardiovasc. Diabetol..

[13] Kishimoto M., Sugiyama T., Osame K., Takarabe D., Okamoto M., Noda M. (2011). Efficacy of ezetimibe as monotherapy or combination therapy in hypercholesterolemic patients with and without diabetes. J. Med. Investig..

[14] Hing Ling P.K., Civeira F., Dan A.G., Hanson M.E., Massaad R., De Tilleghem C.L.B., Milardo C., Triscari J. (2012). Ezetimibe/simvastatin 10/40 mg versus atorvastatin 40 mg in high cardiovascular risk patients with primary hypercholesterolemia: A randomized, double-blind, active-controlled, multicenter study. Lipids Health Dis..

[15] Bangalore S., Kamalakkannan G., Parkar S., Messerli F.H. (2007). Fixed-dose combinations improve medication compliance: A meta-analysis. Am. J. Med..

[16] Fields J., Go J.T., Schulze K.S. (2015). Pill properties that cause dysphagia and treatment failure. Curr. Ther. Res. Clin. Exp..

[17] Arab-Tehrany E., Jacuot M., Gaiani C., Imran M. (2012). Beneficial effects and oxidative stability of omega-3 long-chain polyunsaturated fatty acids. Trends Food Sci. Technol..

[18] Lembke P., Schubert A. (2014). Introduction to fish oil oxidation, oxidation prevention, and oxidation correction. Omega-3 Fatty Acids in Brain and Neurological Health.

[19] Pearnchob N., Dashevsky A., Bodmeier R. (2004). Improvement in the disintegration of shellac-coated soft gelatin capsules in simulated intestinal fluid. J. Control. Release.

[20] Gullapalli R.P., Mazzitelli C.L. (2017). Gelatin and non-gelatin capsule dosage forms. J. Pharm. Sci..

[21] Stoschus B., Allescher H.D. (1993). Drug-induced dysphagia. Dysphagia.

[22] Menditto E., Orlando V., De Rosa G., Minghetti P., Musazzi U.M., Cahir C., Kurczewska-Michalak M., Kardas P., Costa E., Sousa Lobo J.M. (2020). Patient centric pharmaceutical drug product design—The impact on medication adherence. Pharmaceutics.

[23] Porter S., Sackett G., Liu L. (2017). Development, optimization, and scale-up of process parameters: Pan coating. Developing Solid Oral Dosage Forms.

[24] Nazzal S., Wang Y. (2001). Characterization of soft gelatin capsules by thermal analysis. Int. J. Pharm..

[25] HAKATA T., Sato H., Watanabe Y., Matsumoto M. (1994). Effect of storage temperature on the physicochemical properties of soft gelatin capsule shells. Chem. Pharm. Bull..

[26] Manley M. (2014). Near-infrared spectroscopy and hyperspectral imaging: Non-destructive analysis of biological materials. Chem. Soc. Rev..

[27] Jamrógiewicz M. (2012). Application of the near-infrared spectroscopy in the pharmaceutical technology. J. Pharm. Biomed. Anal..

[28] Rosas J.G., Blanco M., González J.M., Alcalà M. (2012). Real-time determination of critical quality attributes using near-infrared spectroscopy: A contribution for Process Analytical Technology (PAT). Talanta.

[29] Wasalathanthri D.P., Rehmann M.S., Song Y., Gu Y., Mi L., Shao C., Chemmalil L., Lee J., Ghose S., Borys M.C. (2020). Technology outlook for real-time quality attribute and process parameter monitoring in biopharmaceutical development—A review. Biotechnol. Bioeng..

[30] Hebbar R., Isloor A., Ismail A. (2017). Contact angle measurements. Membrane Characterization.

[31] Philip A.K., Philip B. (2010). Phase Transited Asymmetric Membrane Capsule: A Means for Achieving Delayed and Controlled Osmotic Flow. Curr. Drug Deliv..

[32] Cole E.T., Scott R.A., Connor A.L., Wilding I.R., Petereit H.U., Schminke C., Beckert T., Cadé D. (2002). Enteric coated HPMC capsules designed to achieve intestinal targeting. Int. J. Pharm..

[33] (2019). Geometrical Product Specifications (GPS)—Surface Texture: Areal—Part 607: Nominal Character-Istics of Non-Contact (Confocal Microscopy) Instruments.

[34] Hu L., Yun D., Gao J., Tang C. (2020). Monitoring and optimizing the surface roughness of high friction exposed aggregate cement concrete in exposure process. Constr. Build. Mater..

[35] Abdi H. (2003). Partial least square regression (PLS regression). Encycl. Res. Methods Soc. Sci..

[36] Zimmermann B., Kohler A. (2013). Optimizing Savitzky–Golay parameters for improving spectral resolution and quantification in infrared spectroscopy. Appl. Spectros..

[37] Guo Q., Wu W., Massart D.I. (1999). The robust normal variate transform for pattern recognition with near-infrared data. Anal. Chim. Acta.

[38] Naharros-Molinero A., Caballo-González M.A., de la Mata F.J., García-Gallego S. (2024). Shell formulation in soft gelatin capsules: Design and characterization. Adv. Healthc. Mater..

[39] Sedighi M. (2023). Encapsulation: Pan-coating. Principles of Biomaterials Encapsulation: Volume One.

[40] Andersson M., Josefson M., Langkilde F.W., Wahlund K.G. (1999). Monitoring of a film coating process for tablets using near infrared reflectance spectrometry. J. Pharm. Biomed. Anal..

[41] Jiao Y., Li Z., Chen X., Fei S. (2020). Preprocessing methods for near-infrared spectrum calibration. J. Chemomet..

[42] Möltgen C.-V., Puchert T., Menezes J.C., Lochmann D., Reich G. (2012). A novel in-line NIR spectroscopy application for the monitoring of tablet film coating in an industrial scale process. Talanta.

[43] Blanco M., Alcalá M. (2006). Content uniformity and tablet hardness testing of intact pharmaceutical tablets by near infrared spectroscopy: A contribution to process analytical technologies. Anal. Chim. Acta.

[44] Roggo Y., Chalus P., Maurer L., Lema-Martinez C., Edmond A., Jent N. (2007). A review of near infrared spectroscopy and chemometrics in pharmaceutical technologies. J. Pharm. Biomed. Anal..

[45] Siepmann J., Peppas N.A. (2012). Modeling of drug release from delivery systems based on hydroxypropyl methylcellulose (HPMC). Adv. Drug Deliv. Rev..

[46] Costa P., Lobo J.M.S. (2001). Modeling and comparison of dissolution profiles. Eur. J. Pharm. Sci..

[47] Merchant H.A., Shoaib H.M., Tazeen J., Yousuf R.I. (2006). Once-daily tablet formulation and in vitro release evaluation of cefpodoxime using hydroxypropyl methylcellulose: A technical note. AAPS PharmSciTech.

